# PP07 One Bad Apple Can Spoil The Barrel: Are We Effectively Evaluating Software As A Medical Device?

**DOI:** 10.1017/S0266462324001806

**Published:** 2025-01-07

**Authors:** Luc Curtis-Gretton, Robert Malcolm

## Abstract

**Introduction:**

There are many differences between medical devices, pharmaceuticals, and Software as a Medical Device (SaMD). This should impact the way SaMDs are evaluated in health technology assessment (HTA). SaMD technologies often target multiple indications, are regularly updated, and often result in non-quantifiable benefits. The objective of this research was to identify problems and potential solutions when evaluating SaMDs in England.

**Methods:**

This research took the perspective of the HTA process in England. We conducted a pragmatic review of publicly available grey literature, such as National Institute for Health and Care Excellence (NICE) guidelines and processes, government schemes, funding mechanisms, and other published reports and opinion pieces, to summarize how SaMDs are currently being evaluated. This included an overview of the current systems and funding structures (inclusive of recent developments), where potential issues may lie, and what is currently being done to address these issues. We concluded by making recommendations to improve the evaluation of these technologies.

**Results:**

Difficulty quantifying outcomes of SaMD technologies, alongside the preference of decision-makers to evaluate technologies for single indications, causes a bottleneck of unevaluated technologies to build. HTA bodies then group many non-identical technologies into single appraisals, resulting in a range of SaMD technologies with varying quality being implemented through managed access agreements. Some schemes and funding mechanisms led by public bodies in England aim to improve efficiency and encourage technological development. However, the HTA process in England remains characterized by long evaluation processes and high clinical evidence requirements, which many SaMD providers find difficult to navigate.

**Conclusions:**

Although progress has been made, there is clear incentive to improve the way in which SaMD technologies are assessed in HTA. We recommend that a more rapid mixed-method approach be implemented. This should draw on quantitative economic analysis supplemented with qualitative clinical, patient, and expert opinion. SaMDs should be evaluated either individually or within smaller groups than current evaluation systems.

